# Research Progress on Effects of Vitamin A on Antioxidant Indicators in Pigs

**DOI:** 10.3390/antiox15101324

**Published:** 2026-10-14

**Authors:** Huijin Jia, Qiwei Liu, Wanghong Zhang, Guangyao Song, Jingrong Feng, Lanlan Yi, Sumei Zhao, Junhong Zhu

**Affiliations:** Yunnan Provincial Key Laboratory of Animal Nutrition and Feed, Faculty of Animal Science and Technology, Yunnan Agricultural University, Kunming 650201, China; 2022210413@stu.ynau.edu.cn (H.J.);

**Keywords:** vitamin A, pigs, oxidative stress, antioxidant markers, antioxidant enzymes, molecular mechanisms, precision nutrition

## Abstract

Oxidative stress constitutes a key restrictive factor that undermines growth performance, reproductive efficiency and meat quality throughout the entire production cycle of commercial pig herds. Vitamin A (VA), an essential fat-soluble nutrient for pigs, fulfills dual antioxidant regulatory functions: it directly scavenges free radicals, and indirectly mediates transcriptional regulation of antioxidant processes via retinoic acid receptor-associated signaling pathways. By integrating recent in vivo feeding trial data and in vitro molecular experimental evidence, this review demonstrates that the antioxidant effect of VA in pigs is characterized by significant stress dependence, dose responsiveness and physiological stage specificity. Under stress challenge conditions, VA can significantly upregulate the body’s antioxidant response, while its regulatory effect is relatively weak under non-stress basal physiological conditions. From the mechanistic perspective, all-trans retinoic acid (ATRA) bound to RAR/RXR receptors can exert synergistic effects with the Nrf2 antioxidant pathway when VA is supplied at physiological nutritional doses, whereas high pharmacological doses of ATRA may induce competitive inhibitory crosstalk. It should be noted that most existing direct promoter-binding evidence is derived from cell line models rather than in vivo porcine tissue samples. For weaned piglets, an optimal dietary VA level can alleviate oxidative damage and intestinal barrier impairment induced by weaning stress, but excessive VA supplementation will trigger lipotoxic oxidative disturbance. In growing-finishing pigs, appropriate VA supplementation can increase glutathione (GSH) content in skeletal muscle and improve the lipid oxidation stability and color stability of pork during post-mortem storage; however, supra-nutritional VA will compete with vitamin E for tissue deposition, which may in turn impair the storage quality of pork. For sows, most existing studies on the reproductive antioxidant effect of VA are derived from intramuscular injection trials, and data from practical dietary VA intervention studies are still insufficient. Existing evidence indicates that maternal VA can regulate the transfer of retinol to offspring via the placenta and colostrum-milk, but no consistent dose-response relationship has been confirmed to date. Meanwhile, relevant research in breeding boars remains extremely insufficient. In terms of nutrient interaction, VA shows antioxidant synergistic effects with vitamin E, vitamin C and selenium, while excessive intake of dietary vitamin D, copper or zinc will reduce the bioavailability of VA and weaken its antioxidant protective effect. Current research in this field is constrained by prominent inter-study heterogeneity, insufficient verification of in vivo molecular mechanisms in pigs, and the absence of antioxidant-targeted VA feeding standards adapted to diverse stress scenarios. The systematic summary of the above findings provides an important reference for the formulation of precision antioxidant nutritional strategies for commercial pig herds. To address the current knowledge gaps, future research should focus on constructing standardized porcine experimental stress models, applying multi-omics technical means to dissect tissue-specific VA antioxidant regulatory networks, clarifying the mechanism of maternal-offspring VA transfer under graded dietary VA intake, and optimizing compatible dosage forms and nutrient combinations adapted to on-farm application scenarios. The advancement of related research will help alleviate production losses caused by oxidative stress and promote the construction of healthy pig production systems.

## 1. Introduction

Under modern intensive large-scale pig production systems, multiple stressors including high-density rearing, temperature stress, nutritional stress, microbial stimulation and reproductive metabolic overload exist persistently, which disrupt the balance between reactive oxygen species (ROS) production and clearance in pig bodies. Excessive accumulated ROS can attack biological macromolecules such as membrane lipids, functional proteins and nucleic acids, further inducing persistent oxidative stress damage in pigs [1]. Oxidative stress can not only inhibit the intestinal development and growth performance of piglets and reduce the antioxidant stability of pork from finishing pigs, but also induce reproductive disorders such as perinatal oxidative imbalance, insufficient lactation and decreased embryo survival rate in sows. It is one of the core factors leading to the reduction in pig production efficiency and the increase in breeding losses [2]. Therefore, adopting nutritional intervention strategies to alleviate oxidative stress and maintain body redox homeostasis has become an important research hotspot and key technical direction in the field of healthy pig production.

Vitamin A (VA) is a category of fat-soluble vitamins with a B-ionone ring and a polyene side chain ring structure. Its naturally occurring active forms mainly include retinol, retinal and all-trans retinoic acid (ATRA), among which ATRA is the core active metabolite that mediates intracellular signal transduction in organisms [3]. Traditional research on animal nutrition has long focused on the functions of VA in regulating growth and development, repairing the mucosal barrier and developing immune organs, while its endogenous antioxidant biological function has long been neglected. With the rapid development of oxidative nutritional biology, a large number of in vivo and in vitro experimental studies have confirmed that, different from traditional non-enzymatic antioxidants, VA has dual functions of direct free radical scavenging and indirect antioxidant regulation at the genetic level, which is a key nutrient connecting nutritional metabolism, oxidative stress and immune regulation [4].

As an essential fat-soluble micronutrient for swine, VA plays a critical role in modern pork production. In addition to its well-characterized classical functions in supporting visual development, maintaining epithelial tissue integrity and regulating immune function, a growing body of production-based evidence shows that the nutritional status of VA profoundly affects stress resistance, pork quality, reproductive performance of sows and growth performance in commercial pig herds. Improper VA supplementation, whether deficiency or excess intake, often leads to oxidative dysregulation, shortened shelf life of pork, increased morbidity and reduced reproductive efficiency, which ultimately causes considerable economic losses to the swine industry. However, current research on VA-mediated antioxidant regulation in pigs is still fragmented.

To date, a large number of experimental datasets on the regulatory role of VA in the antioxidant function of pigs have been accumulated globally. However, existing studies still face multiple key problems: the research results have high heterogeneity, the regulatory characteristics of VA in pigs at different physiological stages are not clear, the dose-effect mechanism has not been fully elucidated, and the core molecular regulatory pathway network remains ambiguous. Furthermore, there is still a lack of systematic and in-depth review literature to integrate and summarize the existing research findings. Based on this research background, this paper integrates the research outputs published in authoritative academic journals at home and abroad in the past, systematically clarifies the molecular mechanism of VA regulating redox homeostasis in pigs, quantitatively summarizes and analyzes the influence rule of VA on antioxidant indexes in different tissues of pigs at different growth stages, identifies the key regulatory factors, sorts out the existing academic controversies and technical bottlenecks, and prospects the future research directions, so as to provide a theoretical basis for the scientific application of VA in precise antioxidant nutritional regulation of swine.

## 2. The Molecular Mechanisms Underlying VA Regulation of Oxidative Homeostasis in Pigs

The regulation of the antioxidant system in porcine organisms by VA does not follow a single molecular mechanism. Instead, VA achieves multi-pathway synergistic regulation through physical antioxidant effects, chemical free radical quenching, transcriptional-level gene regulation and coordinated modulation of metabolic pathways. Its core regulatory mechanisms can be divided into two major systems: direct antioxidant effects and indirect signal-mediated regulatory effects. These two mechanisms complement each other and jointly maintain the redox homeostasis of the organism.

### 2.1. Direct Antioxidant Effect: Scavenging Reactive Oxygen Species and Stabilizing Biological Membrane Structure

The direct antioxidant activity of vitamin A is primarily mediated by free retinol, a function that is highly dependent on its molecular scaffold containing a long-chain continuous conjugated unsaturated polyene double bond structure; this polyene chain possesses a delocalized electron system and high electron affinity, enabling it to directly capture and neutralize various toxic ROS such as superoxide anion (O_2_^−^), hydroxyl radical (·OH), lipid peroxyl radical, and singlet oxygen through reversible hydrogen donation and electron transfer reactions. This mechanism rapidly terminates the lipid peroxidation chain reaction mediated by polyunsaturated fatty acids in cell membranes, thereby blocking the cascade pathway through which oxidative damage progressively amplifies from membrane lipids to intracellular proteins, DNA, and organelles, making vitamin A an efficient chain-breaking antioxidant in lipid-rich environments [5,6]. In contrast to water-soluble ascorbic acid, which can only transiently scavenge ROS within the aqueous cytoplasmic compartment, retinol is a highly lipophilic micronutrient that can be specifically inserted into the hydrophobic domain of the phospholipid bilayer of cell membranes via its hydrophobic tail. Through van der Waals forces and hydrophobic interactions with membrane lipids including phosphatidylcholine and phosphatidylethanolamine, retinol stabilizes the spatial conformation of both membrane proteins and phospholipids, decreases the fluidity and oxidative permeability of membrane lipids, and inhibits the diffusion of ROS and lipid peroxides into intracellular organelles such as mitochondria and endoplasmic reticulum. This mechanism significantly attenuates oxidative stress-induced reduction in mitochondrial membrane potential, endoplasmic reticulum stress and downstream apoptotic cascade reactions, thereby alleviating oxidative damage and secondary inflammatory injury in livestock and poultry tissues [7]. In terms of tissue distribution, retinol can persist for a long duration in multiple lipid-enriched tissues (including adipose tissue, germ cells, skeletal muscle and hepatocyte membrane systems and continuously exerts membrane protection and free radical scavenging effects. Compared with water-soluble antioxidants that only exist transiently in the aqueous cytoplasm, dietary retinol acts as the first physicochemical synergistic defense barrier for the cellular membrane system against oxidative attack, providing sustained protection against ROS-induced damage caused by common breeding stresses such as weaning, transportation and high temperature [8]. It should be noted that the direct antioxidant activity of retinol is environment-dependent: its free radical quenching activity reaches the optimal level under the low oxygen partial pressure in physiological tissues, while retinol is prone to autoxidation in high-oxygen environments, which reduces its direct antioxidant efficiency. Under such circumstances, retinol needs to act synergistically with VE and endogenous glutathione (GSH) to construct a complete lipid-water biphasic antioxidant defense system [9].

### 2.2. Indirect Antioxidant Activity: Regulation of Antioxidant Gene Expression Mediated by the Retinoic Acid Receptor Signaling Pathway

The core antioxidant function of retinol is highly dependent on the transcriptional regulatory pathway mediated by its active metabolite all-trans retinoic acid (ATRA). This gene-level regulatory mechanism is the most essential feature distinguishing retinol from other antioxidant vitamins such as vitamin E and vitamin C, which exert antioxidant effects only through direct molecular scavenging of free radicals [10]. Retinol undergoes two-step oxidation catalyzed by intracellular alcohol dehydrogenase and aldehyde dehydrogenase to generate ATRA. ATRA has nuclear targeting ability and can penetrate the nuclear membrane to specifically bind to nuclear retinoic acid receptors (RARα/β/γ) and retinoid X receptors (RXRα/β/γ), forming heterodimer receptor-ligand complexes. These complexes can specifically recognize and targetedly bind to the conserved retinoic acid response elements (RARE) located in the promoter region of multiple antioxidant genes, recruit transcriptional coactivators, and positively promote the transcription initiation of core antioxidant-related coding genes after chromatin remodeling and opening, continuously upregulate the synthesis levels of antioxidant enzymes and non-enzymatic antioxidant molecules, and thereby establish a long-lasting endogenous antioxidant defense mechanism [11,12].

In the porcine metabolic system, classical genes encoding antioxidant functions are categorized into two major groups. The first group encompasses genes encoding antioxidant enzymes, which primarily include cytosolic superoxide dismutase 1 gene (Superoxide Dismutase 1, *SOD1*), glutathione peroxidase 1 gene (Glutathione peroxidase 1, *GPX1*), and catalase gene (Catalase, *CAT*). The second group comprises non-enzymatic antioxidant regulatory genes that participate in the synthesis and catabolic regeneration of GSH, directly determine the total intracellular content of reduced GSH, and act as critical substrates for the maintenance of cellular redox homeostasis [13]. Large-scale in vivo fattening trials covering piglets, finishing pigs and pregnant sows, combined with in vitro experimental models based on porcine primary intestinal epithelial cells and hepatocytes, consistently confirm that appropriate dietary supplementation doses of ATRA in vivo, or suitable ATRA concentrations in vitro, can significantly upregulate both the mRNA abundance and protein expression level of key genes associated with *SOD1*, *GPX1*, *CAT* and *GSH* synthesis in porcine liver, small intestinal mucosa, skeletal muscle and ovarian tissues [14]. Meanwhile, phenotypic measurements of experimental animals show that the activities of total superoxide dismutase (T-SOD), glutathione peroxidase (GSH-Px) and catalase (CAT) increase significantly, and the total antioxidant capacity (T-AOC) of the organism is substantially improved. Furthermore, the capacity and efficiency of the endogenous antioxidant system to scavenge excess ROS are significantly enhanced, which effectively alleviates systemic oxidative damage induced by weaning stress, high temperature stress and transportation stress [15,16].

In addition to directly activating the transcription of antioxidant genes, ATRA can also inhibit lipid peroxidation by targeting the source of oxidative damage, thereby constructing a bidirectional regulatory network for cellular oxidative homeostasis. Via transcriptional regulatory mechanisms, ATRA negatively regulates both the gene expression and protein activity of lipoxygenases (LOX), which are the key rate-limiting enzymes in the lipid peroxidation process. This regulatory effect reduces the initiation of oxidative degradation of polyunsaturated fatty acids and further decreases the generation rate of lipid peroxides. Meanwhile, ATRA suppresses the accumulation of downstream cytotoxic lipid oxidation end products including malondialdehyde (MDA) and 4-hydroxynonenal (4-HNE), thereby alleviating mitochondrial dysfunction, DNA oxidative mutation and cell apoptosis induced by lipid peroxides [17]. Existing studies have confirmed that the antioxidant effect of ATRA mediated by transcriptional regulation shows a significant dose-dependent characteristic: low concentrations of ATRA cannot effectively activate the retinoic acid receptor-retinoid X receptor (RAR/RXR) signaling pathway, leading to only limited upregulation of antioxidant gene expression [18]; excessive ATRA disrupts intracellular retinol homeostasis, induces lipotoxicity and oxidative imbalance, and ultimately reduces the systemic antioxidant capacity of the organism. Only when ATRA is maintained within a suitable concentration range can it simultaneously exert dual protective effects by activating antioxidant genes and inhibiting lipid peroxidation [18,19].

From the perspective of pathway crosstalk, ATRA-mediated retinoic acid receptor (RAR) signaling can exert synergistic interactions with the Kelch-like epichlorohydrin-associated protein 1-nuclear factor erythroid 2-related factor 2 (Keap1-Nrf2) antioxidant signaling pathway: ATRA upregulates the protein expression of Nrf2 and facilitates the nuclear translocation of Nrf2; the two transcription factors subsequently bind to the promoter regions of antioxidant response genes, which further upregulates the transcriptional abundance of antioxidant genes including *GPX1* and *SOD1*, ultimately forming an endogenous dual antioxidant regulatory axis integrating retinoic acid signaling and Nrf2 signaling. Notably, this crosstalk between signaling pathways presents obvious context-dependent characteristics. Under pharmacological high-dose ATRA exposure, activated RAR-RXR heterodimers may either compete for the shared RXR cofactor or directly interact with the Nrf2 protein, thereby exerting an inhibitory effect on the transcription of antioxidant response element (ARE)-driven target genes [17,20]. This inhibitory regulatory pattern has been frequently confirmed in tumor-derived cell line models, while such inhibitory effects are rarely reported in in vivo porcine stress models under physiological nutritional doses of VA. This molecular mechanism constitutes the core theoretical basis for why VA exhibits superior antioxidant efficacy relative to single exogenous antioxidants in swine [21,22]. It should be noted that the majority of direct transcriptional evidence supporting the ATRA-RAR/RXR-dependent upregulation of *GPX1* and *CAT* is derived from in vitro cell models. Consistent phenotypic observations from in vivo feeding trials in swine have confirmed that appropriate VA supplementation significantly elevates the activities of CAT and GSH-Px in serum and tissue samples across weaned piglets, growing-finishing pigs and sows. However, direct in vivo molecular evidence verifying the binding of RAR-RXR to the promoter regions of porcine CAT and *GPX1* genes remains insufficient. The increased activity of antioxidant enzymes detected in vivo may also result from indirect secondary effects, including VA-mediated inhibition of inflammatory cascades, improvement of intestinal barrier function, modulation of trace element status (e.g., selenium bioavailability) and crosstalk with other nuclear receptor signaling pathways. Accordingly, the elevated activity of CAT and GSH-Px observed in VA-supplemented pigs cannot be solely attributed to the direct transcriptional activation driven by the ATRA-RAR/RXR complex, and multiple potential indirect regulatory pathways should be considered when interpreting in vivo experimental results.

### 2.3. Cross-Regulation: Inhibition of Inflammation-Mediated Secondary Oxidative Stress

**Figure 1:**
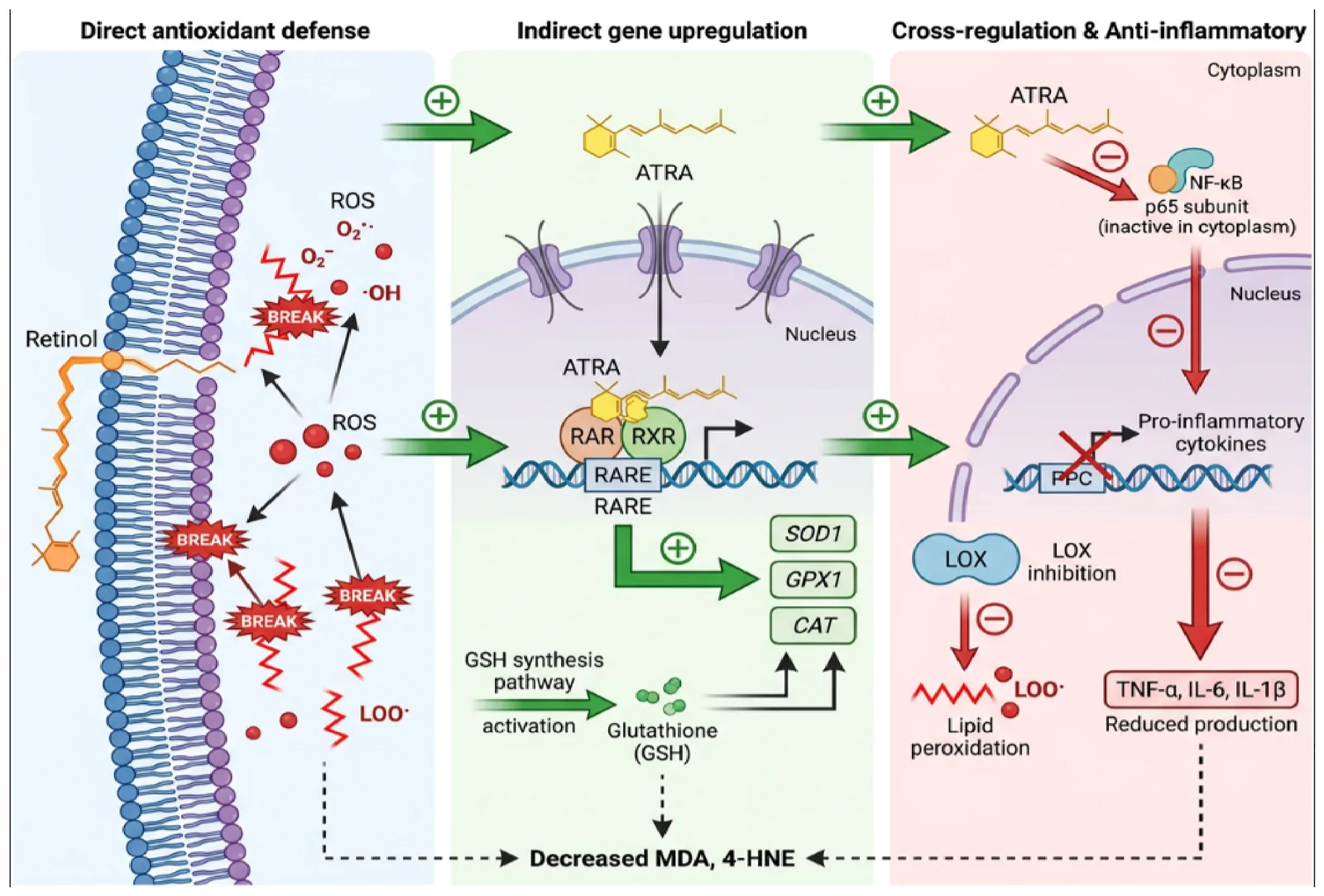
Schematic diagram of the antioxidant mechanism of VA. Black solid arrows represent promoting effects; red solid arrows indicate inhibitory effects; green solid arrows indicate signal transduction/translocation processes.

In the organism of animals, oxidative stress and inflammatory response form a tightly coupled positive feedback loop, in which the two processes mutually promote each other and continuously amplify the resulting damaging effect: On the one hand, when the organism is subjected to oxidative stress, a large amount of ROS accumulates in tissues, which directly activates the canonical nuclear factor-kappa B (NF-κB) inflammatory signaling pathway and initiates inflammation-related transcriptional programs; On the other hand, after the activation of the NF-κB pathway, a variety of pro-inflammatory cytokines are synthesized and released, which further induce organelles such as mitochondria to generate additional ROS. This process drives the sustained massive production of ROS in the organism, and ultimately aggravates oxidative damage and functional disorder of host tissues [23]. The active metabolite of vitamin A, all-trans retinoic acid, can exert cytoprotective effects by mediating the all-trans retinoic acid-retinoic acid receptor (ATRA-RAR) signaling pathway. It directly inhibits the nuclear translocation of the NF-κB p65 subunit in swine, thereby reducing the transcriptional activation of downstream inflammatory genes mediated by this transcription factor. This mechanism effectively reduces the synthesis and secretion of pro-inflammatory factors including tumor necrosis factor-alpha (TNF-α), interleukin-6 (IL-6) and interleukin-1β (IL-1β), and thus blocks the vicious cycle between inflammation and oxidative stress at the initial stage of inflammatory response [24]. This cross-regulatory mechanism, which simultaneously targets the core oxidative stress pathway and the inflammatory coupling interface, is a key pathway through which vitamin A effectively alleviates intestinal oxidative stress injury in weaning piglets and improves the metabolic stress status of perinatal sows. It also constitutes an important functional advantage that distinguishes the efficacy of vitamin A from that of single exogenous antioxidants that only target the oxidative stress pathway. The multi-layer antioxidant regulatory mechanism of VA described above is summarized in Figure 1.

## 3. Differential Regulatory Effects of VA on Antioxidant Indices in Pigs Across Different Physiological Stages

In pigs, the oxidative metabolic capacity, stress sensitivity, and nutrient utilization efficiency of VA differ significantly across different physiological stages. Accordingly, the magnitude of VA’s regulatory effect on antioxidant parameters, as well as its functional targets and optimal dosages, shows clear stage-specific characteristics among weaned piglets, growing-finishing pigs, and breeding sows. The specific regulatory mechanisms are elaborated as follows.

### 3.1. Relieves Weaning Stress and Enhances the Body’s Antioxidant Capacity

Weaned piglets constitute the physiological stage wherein the antioxidant regulatory effects of VA are most prominent, and represent the core focus of current research on VA antioxidant functionality in the field of animal nutrition. After weaning, commercial pig production requires piglets to transition from suckling maternal milk to independent intake of solid feed; during this transition, piglets must not only adapt to dramatic changes in dietary texture and nutrient composition, but also tolerate multiple combined stressors including intestinal microbiota perturbation, pen mixing, and rearing environment changes. These stressors ultimately trigger explosive accumulation of ROS in piglets in vivo [25]. As a result, the function of the endogenous antioxidant system, which is normally maintained via coordinated enzymatic and non-enzymatic pathways, decreases significantly. This is reflected in quantifiable antioxidant indicators: the activities of T-SOD, GSH-Px, and T-AOC in serum typically decrease substantially, while the content of the lipid peroxidation product MDA increases significantly. This disrupted redox status further induces a series of production problems, such as intestinal barrier damage, increased diarrhea incidence, and growth retardation in weaned piglets [26]. At this stage, the various physiological functions of piglets remain under development. The endogenous antioxidant system, which is responsible for ROS scavenging and oxidative homeostasis maintenance, has not yet fully developed and matured, resulting in inherently low basal endogenous antioxidant capacity. Accordingly, weaned piglets exhibit high sensitivity to nutritional regulation mediated by exogenous VA [27]. Efficacy trials with gradient VA supplementation levels further verified that VA regulation of the antioxidant system in weaned piglets follows a typical “low-dose promotion, high-dose inhibition” dose-response pattern: the threshold of 2200 IU/kg, the optimal dosage of 4400 IU/kg, and the upper limit of 17,600 IU/kg mentioned in this section are obtained from existing pig nutritional requirement standards and relevant feeding trials on piglets. [4]. When dietary VA supplementation is below 2200 IU/kg, VA cannot effectively activate the intracellular ATRA signaling pathway, and thus no significant antioxidant regulatory effect is observed; 4400 IU/kg is the optimal supplementation level for this physiological stage, achieving a synergistic optimum that simultaneously maximizes antioxidant function improvement and growth performance promotion; however, when VA supplementation exceeds 17,600 IU/kg, excess VA (a fat-soluble vitamin) accumulates in vivo, induces toxic effects and disrupts the original redox balance of the organism, ultimately reducing the activities of various antioxidant enzymes and increasing MDA concentration, which in turn exacerbates oxidative damage to the body [4]. Supplementing 4400 IU/kg VA in the diet (the currently industry-recognized optimal recommended dose) can increase the activities of CAT and GSH-Px in the serum of weaned piglets, significantly reduce serum MDA content, and improve intestinal T-AOC activity [28]. Notably, VA exerts obvious index-specific regulatory characteristics on different antioxidant parameters of weaned piglets: at the optimal dosage, VA does not significantly affect T-SOD activity in weaned piglets, and only specifically activates the two core antioxidant enzyme pathways of CAT and GSH-Px. This conclusion has been widely verified in existing research [29]. Furthermore, the coated VA products currently developed by the industry adopt a special coating structure, which reduces the loss of VA activity caused by high temperature during feed pelleting and degradation by gastric acid in piglets. The >30% increase in the bioavailability of coated VA was derived from in vivo gastrointestinal digestion and absorption experiments in pigs [30]. Therefore, the overall bioavailability of coated VA is more than 30% higher than that of conventional VA products, and its antioxidant efficacy has higher stability in actual production, which can effectively improve the intestinal oxidative stress status of piglets and reduce the incidence of post-weaning diarrhea [30].

### 3.2. Targetedly Improves Meat Quality Antioxidant Capacity and Alleviates Environmental Stress-Induced Damage

Fattening pigs, as the cohort representing the terminal weight-gain phase in the swine production cycle, have fully developed physiological functions; their capacity for metabolic regulation and maintenance of internal homeostasis is also more stable. Under conventional standardized feeding conditions, their baseline oxidative stress levels remain consistently low. Consequently, additional supplementation of exogenous vitamin A does not exert a significant regulatory effect on the core baseline antioxidant parameters in their serum [31]. The antioxidant function of vitamin A at this stage primarily focuses on two key aspects: antioxidant protection of muscle tissue and mitigation of damage induced by adverse environmental stressors. Under normal farming conditions that comply with industry standards, the addition of vitamin A to the diet can significantly reduce MDA levels in the representative longissimus dorsi muscle of fattening pigs, while effectively increasing GSH levels in muscle tissue and enhancing overall antioxidant stability. This approach inhibits lipid oxidation reactions occurring in muscle tissue during post-harvest storage at the source, delays the deterioration of muscle color, reduces water loss during storage and processing, and ultimately extends the shelf life of pork products-thereby significantly improving meat quality after the slaughter of fattening pigs from multiple perspectives [32]. The molecular mechanism underlying increased muscle GSH concentration induced by dietary VA supplementation has not been fully verified in porcine muscle tissue. On the basis of existing in vitro and cross-species evidence, two plausible mechanistic pathways have been proposed: (1) ATRA activates the Nrf2 signaling axis, further upregulates the transcription of GCLC and GCLM, the rate-limiting genes associated with GSH synthesis, thereby promoting de novo GSH biosynthesis in muscle cells; (2) VA-mediated inhibition of lipid peroxidation and inflammatory responses reduces GSH consumption caused by oxidative stress damage. However, direct molecular evidence from porcine skeletal muscle to support either pathway remains insufficient. Furthermore, there is a non-linear dose-dependent relationship between dietary VA supplementation dose, retinol accumulation in pork, lipid oxidation stability after slaughter, and meat color stability during storage. Moderate dietary VA supplementation increases retinol deposition in muscle and adipose tissue, which may help alleviate post-mortem lipid peroxidation and delay myoglobin oxidation-induced meat discoloration. In contrast, excessive supranutritional VA intake competes with vitamin E for tissue deposition, reduces α-tocopherol reserves in muscle, and unexpectedly increases the susceptibility of muscle to lipid oxidation. To date, a unified quantitative dose threshold that associates dietary VA levels, pork retinol concentration and meat storage quality indicators has not been established for finishing pig production. When pigs are subjected to adverse stress conditions-such as heat stress or high-density farming-their endogenous antioxidant system is significantly suppressed; under these circumstances, the regulatory effect of VA on the antioxidant system becomes markedly activated. VA can reverse the decline in SOD and GSH-Px activity induced by heat stress, reduce the accumulation of ROS in serum and muscle tissue, help maintain homeostasis within the growth and metabolic processes of the animals, and mitigate the reduction in daily weight gain observed in fattening pigs under stressful conditions [33]. In sows subjected to summer heat stress (characterized by a high temperature-humidity index), serum SOD activity decreases at parturition, and serum GSH-Px activity declines significantly on the 14th day postpartum, which is accompanied by aggravated lipid peroxidation [34]. Under non-heat-stress conditions during late gestation, dietary supplementation with β-carotene (provitamin A) can increase serum GSH-Px activity in sows by approximately 29–31% relative to the control group. However, compared with the relatively abundant research on finishing pigs, specialized in vivo feeding trials evaluating the effects of graded dietary VA dosages in heat-stressed sows are still insufficient at present [1,35]. Additionally, VA can modulate the organism’s lipid oxidation metabolism pathways, thereby reducing abnormal deposition of oxidized lipids in fattening pigs and alleviating oxidative stress-mediated lipid metabolism disorders, ultimately enhancing the uniformity and production stability of the market-ready livestock population. Long-term, moderate supplementation of VA throughout the entire fattening period can also upregulate the baseline expression levels of antioxidant-related genes in muscle tissue and gradually increase the overall antioxidant reserve capacity of the animal, fundamentally enhancing the resilience of fattening pigs to various adverse environmental stresses [36]. Current research on the addition of vitamin A to the diets of sows remains relatively limited. Existing studies have demonstrated that appropriate maternal vitamin A supplementation can regulate the transplacental and colostrum/milk transport of vitamin A, thereby potentially influencing the antioxidant status, immune development, and early growth performance of the offspring. However, there is still a significant scarcity of dose-response data regarding the relationship between vitamin A levels in sow diets and the physiological phenotypes of their offspring, and findings across different studies remain inconsistent. Further systematic feeding studies are therefore warranted to validate these findings.

### 3.3. Regulates Perinatal Oxidative Homeostasis and Improves Reproductive Antioxidant Status

During the three discrete physiological stages of estrus, gestation and lactation, sows encounter physiological oxidative stress with distinct stage-specific characteristics, with the most severe oxidative stress observed in the peripartum period (defined as the interval from 7 days before parturition to 7 days after parturition) [37]. The peripartum period is a critical stage in the reproductive cycle of sows, featuring the most dramatic physiological fluctuations throughout the entire reproductive process. Throughout this stage, sows complete extensive physiological preparation for farrowing and lactation, during which the systemic metabolic rate increases sharply within a short timeframe, resulting in rapid consumption of large quantities of energy. As a consequence, the process of mitochondrial energy metabolism in somatic cells produces excessive ROS, which readily disrupt the overall redox homeostasis of the organism. This oxidative imbalance is a core contributing factor leading to postpartum weakness, insufficient lactation and low litter embryo survival rate in sows [38]. As a well-recognized key regulatory factor in sow reproductive nutrition, VA can systematically ameliorate the antioxidant status of sows at multiple levels across all reproductive stages [39,40,41].

### 3.4. Antioxidant Regulation in Breeding Boars

**Figure 2:**
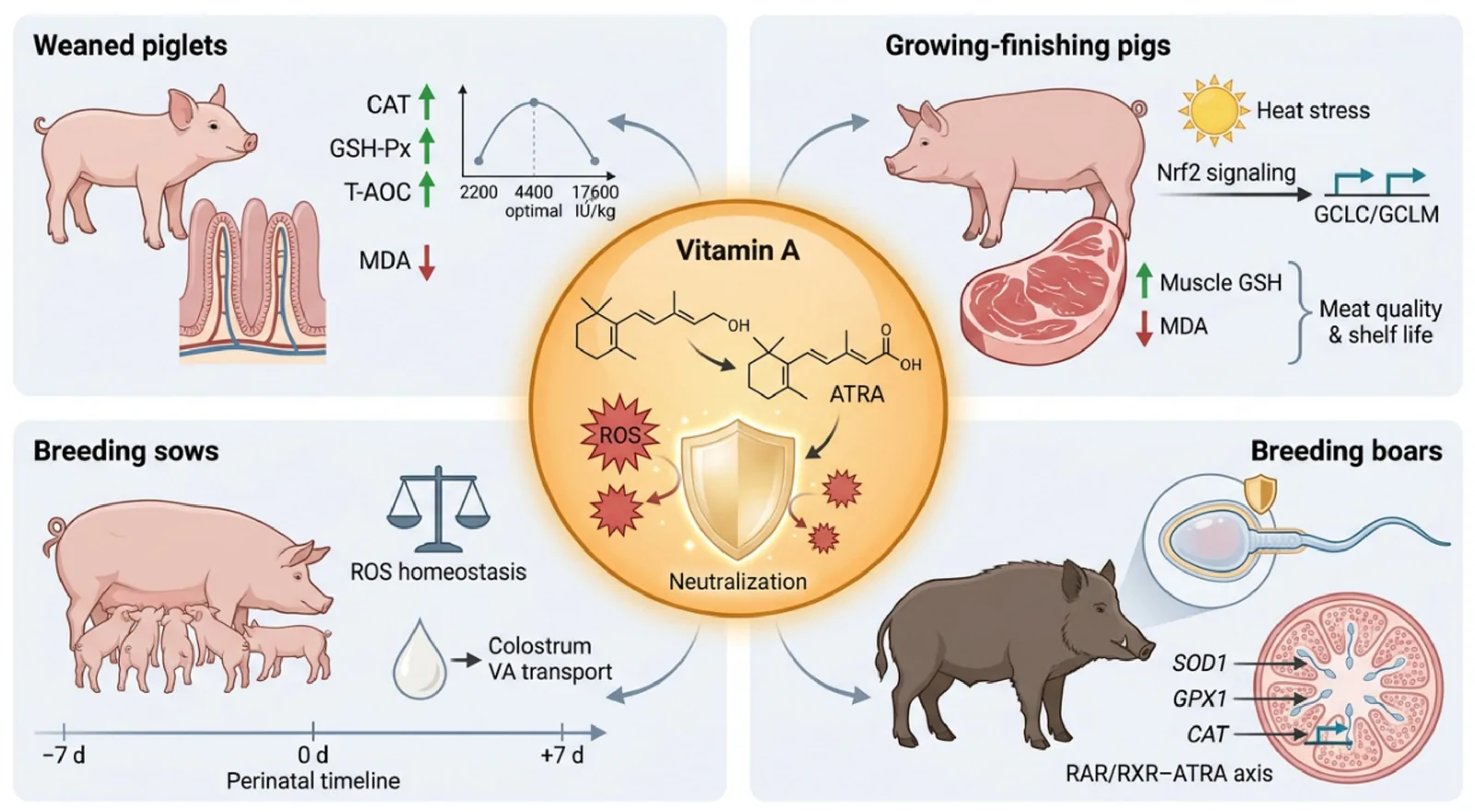
Schematic diagram illustrating the differential regulatory effect of VA on antioxidant biomarkers in pigs at different physiological stages. Green solid arrows represent promoting effects; red solid arrows indicate inhibitory effects; black solid arrows indicate signal transduction/translocation processes; blue solid arrows denote gene transcription and expression.

Adult breeding boars are subjected to long-term high reproductive metabolic loads, and the process of spermatogenesis is highly vulnerable to oxidative damage induced by ROS [42]. Excessive ROS can attack the sperm plasma membrane which is rich in polyunsaturated fatty acids, initiate lipid peroxidation, increase MDA concentration in seminal plasma, impair sperm DNA integrity, reduce sperm motility, and ultimately degrade boar semen quality and fertilization capability [43]. VA mediates the maintenance of testicular redox homeostasis in breeding boars through dual direct and indirect antioxidant pathways. Specifically, retinol can integrate into the membranes of sperm and Sertoli cells to directly scavenge lipid-soluble free radicals; meanwhile, ATRA activates the RAR/RXR signaling axis in the testis, upregulates the transcriptional expression of *SOD1*, *GPX1* and *CAT* in testicular tissue and seminal plasma and enhances T-AOC and GSH-Px activity in semen [44]. At present, the published research on the antioxidant effects of VA in breeding boars remains relatively insufficient. Existing studies lack systematic gradient dose trials and multi-omics evidence based on testicular tissue, and no unified industrial standard has been established for the optimal dietary VA supplementation dose for breeding boars across different ages and stress scenarios, which requires further in-depth investigation in future research. The differential regulatory effects of VA on antioxidant indicators across weaned piglets, finishing pigs and sows are summarized in Figure 2.

## 4. Factors Influencing the Antioxidant Regulatory Effect of VA

### 4.1. Addition Dosage and Dosage Form

The dosage of VA is the core variable that determines the antioxidant efficacy of VA in pigs. Pigs at different growth and reproductive stages exhibit different demands for the antioxidant effects of VA, resulting in significant differences in the optimal dosage of VA required to exert antioxidant effects in each stage. Moreover, this dose-effect relationship strictly follows the fundamental principle that appropriate doses activate antioxidant pathways, while excessive doses induce oxidative toxicity [45]. In addition to dosage, the formulation of VA supplements directly determines the bioavailability of VA and the duration of its action in pigs. Conventional crystalline VA has extremely poor chemical stability: during the high-temperature granulation process of feed production, storage at ambient temperature in farms, and exposure to the acidic environment of the pig stomach, the activity of crystalline VA will decrease significantly, and the total activity loss rate can exceed 40%, which ultimately leads to extremely weak antioxidant effects [46]. In contrast, the sustained-release VA formulation prepared by the starch-gelatin double-layer coating process can resist the harsh environment of high-temperature granulation and the degradation effect of gastric acid. The effective release rate of VA after the preparation reaches the intestinal tract is significantly improved, which can achieve stable and long-lasting antioxidant regulation in pigs [47]. As a new type of VA preparation, ATRA-active preparation can directly act on specific nuclear receptors without in vivo metabolic transformation and has a faster onset of action. Therefore, it is very suitable for rapid antioxidant intervention during acute stress and can effectively deal with sudden oxidative stress in weaned piglets or perinatal sows [48].

### 4.2. Stress Response in Swine Production

The antioxidant regulatory effect of VA is strictly stress-dependent, and its regulatory efficacy differs significantly across distinct physiological states of pigs. Under normal non-stressed basal maintenance conditions, the endogenous antioxidant system of pigs can effectively maintain basal redox homeostasis. In this scenario, additional exogenous VA supplementation only slightly improves the baseline antioxidant indicators of the body and fails to produce significant additional antioxidant benefits. However, when pigs are exposed to various oxidative stress stimuli such as weaning stress, heat stress, reproductive stress and pathogenic infection, the internal homeostasis of the body is disrupted, the endogenous antioxidant enzyme system that maintains normal function is significantly inhibited, and a large amount of ROS accumulates in the body. Under this condition, the ATRA signaling pathway mediated by VA is specifically activated, which greatly amplifies the antioxidant benefit of exogenous VA supplementation. Under oxidative challenge induced by NO, pre-treatment with VA increased GPx activity by 42–58% compared with the stress-only group. In contrast, VA supplementation only caused marginal, non-significant changes in antioxidant enzyme activities in non-stressed control cells [49]. This degree of enhancement was observed in an in vitro cell culture model and cannot be directly extrapolated to in vivo physiological responses of pigs. The distinct functional characteristic confirms that VA is an indispensable key functional nutrient in the stress prevention and control strategy of pig production.

### 4.3. Synergistic and Antagonistic Interactions Between Nutrients

VA exerts a highly significant synergistic antioxidant effect with other antioxidant substances. VA can regulate the expression of antioxidant-related genes: it participates in scavenging free radicals on cell membranes in synergy with VE; it promotes the regeneration of oxidized VE when acting in conjunction with VC; and it activates the activity of the core antioxidant enzyme GSH-Px in combination with selenium. These four types of antioxidant-active substances coordinate with each other to construct a complete closed-loop endogenous antioxidant circulation system, which can comprehensively improve the overall antioxidant capacity of pigs at multiple levels, including gene expression regulation, direct free radical scavenging and key component activation in the antioxidant system [29]. Furthermore, VA can repair damaged intestinal epithelial barriers, optimize the composition of intestinal microbiota in pigs, and reduce the generation of secondary oxidative stress mediated by intestinal inflammation. Therefore, when VA is used in combination with probiotics, plant extracts and anti-inflammatory feed additives, it can form a more systematic synergistic antioxidant regulatory effect [50].

**Figure 3:**
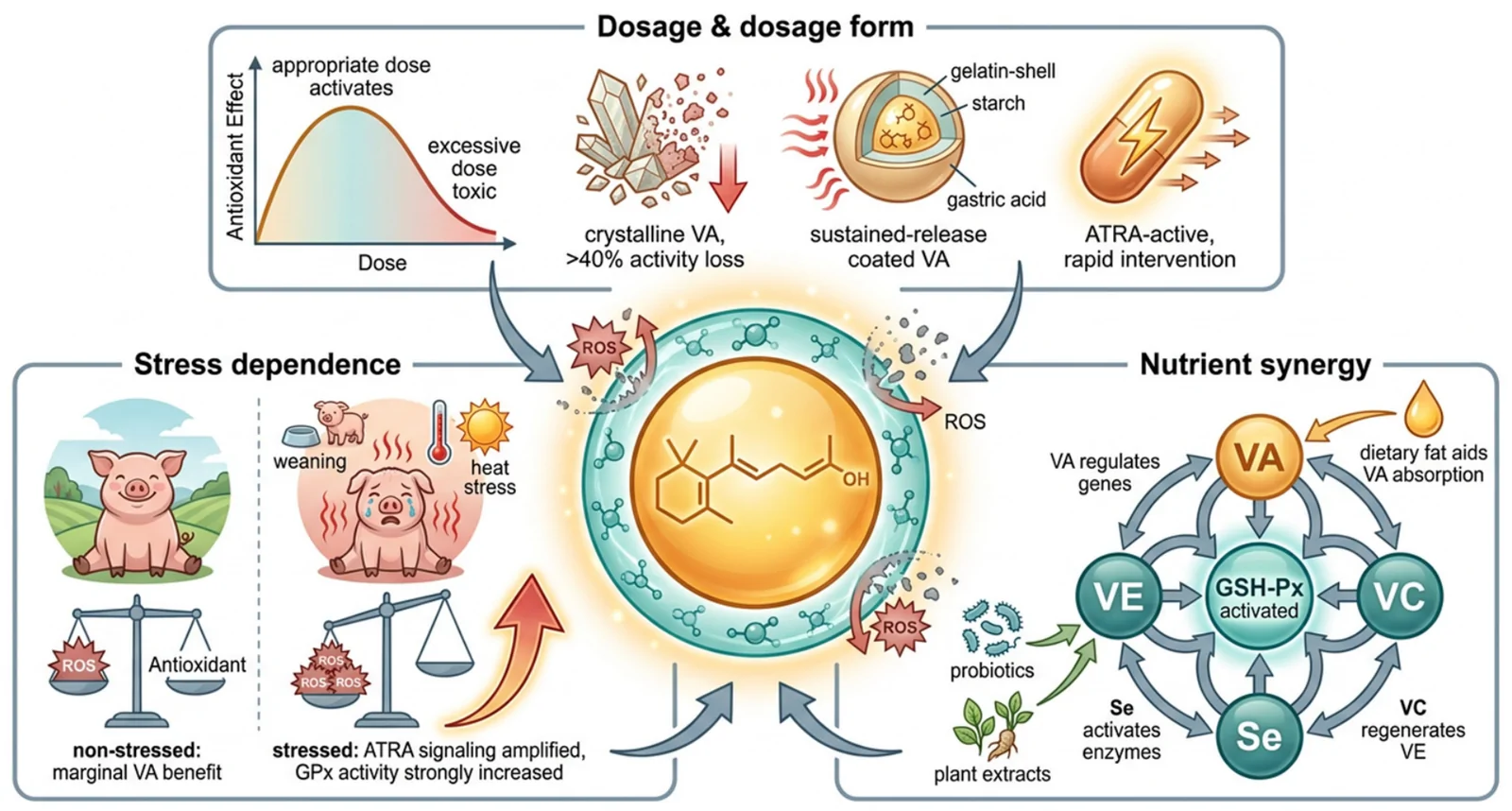
Schematic summary of key factors modulating vitamin A-mediated antioxidant responses in pigs, including dosage, formulation, stress status and nutrient interactions. Grey unidirectional arrows indicate regulatory effects of external factors on vitamin A signaling; orange arrows represent promoting or amplifying effects; red arrows denote ROS generation and oxidative insult; grey bidirectional arrows indicate synergistic interactions among nutrients.

Existing nutritional compatibility studies in breeding sows have confirmed that VA, VE and selenium exhibit a remarkably significant reproductive antioxidant synergistic effect. Specifically, VA mainly regulates the expression of antioxidant-related genes in vivo; VE primarily provides direct protective effects for cell membranes; while selenium is responsible for activating the activity of endogenous antioxidant enzymes. The functions of the three nutrients are fully complementary, which can significantly improve the overall reproductive stress resistance of breeding sows. At present, this combination scheme has been recognized by the industry as a core nutritional strategy in the nutritional management of perinatal sows [51]. In addition, as a fat-soluble vitamin, the intestinal absorption and transport process of VA will be impaired when dietary fat content is insufficient, which hinders the effective accumulation of VA in pig tissues and ultimately leads to a significant decline in its antioxidant activity [52]. Multiple intrinsic and extrinsic factors modulate the antioxidant effects of dietary VA in pigs, and these factors are summarized in Figure 3.

## 5. Deficiencies in Existing Research and Future Research Prospects

Currently, a consensus has not yet been reached among existing studies on the regulatory effects of VA on antioxidant-related parameters in pigs. The heterogeneity observed in current research findings arises from inconsistent settings across different experimental systems, particularly with regard to pig breeds, experimental age, basal diet composition, stress model construction, and the time point of antioxidant parameter determination. Moreover, the absence of standardized experimental protocols in the current research field further impedes the formation of a unified scientific understanding of the antioxidant effect of VA. At present, most studies on the regulatory role of VA in porcine antioxidant function remain at the stage of phenotypic indicator observation and statistical analysis, and the elucidation of core molecular regulatory mechanisms is insufficiently in-depth. Although multiple in vivo feeding trials have reported that VA supplementation elevates the activities of CAT and GSH-Px in pigs across diverse physiological stages, direct in vivo molecular evidence verifying that the ATRA-RAR/RXR complex directly targets the promoters of porcine *CAT* and *GPX1* genes remains insufficient to date. The observed alterations in enzyme activity may be confounded by indirect biological effects, including anti-inflammatory responses, improvement of intestinal barrier function, and changes to trace element metabolism, which have not been systematically resolved in porcine experimental models. For growing-finishing pigs, the molecular pathways underlying VA-induced elevation of muscle GSH content still require further experimental verification in porcine skeletal muscle. Furthermore, the quantitative dose-response relationships linking dietary VA dosage, retinol deposition in pork, lipid oxidation status, and meat color stability under cold storage conditions have not been systematically characterized, which impedes the development of precise nutritional strategies for extending the shelf life of pork. Furthermore, the dose-dependent effects of maternal dietary VA on placental retinol transport, retinol secretion in colostrum and milk, as well as the antioxidant status and developmental phenotypes of the offspring, still require systematic in-depth investigation. To date, it has only been confirmed that RAR/RXR is the core receptor pathway through which VA exerts antioxidant regulatory effects [18]. However, key scientific issues including the tissue-specific regulatory characteristics of receptor subtypes, the cross-regulatory mechanism of downstream pathways, and the systematic analysis based on multi-omics technology have not yet been resolved, and corresponding mechanistic verification data at the protein and metabolic levels are still lacking. In the current pig production industry, the dietary supplementation standard of VA is still formulated mainly based on growth performance indicators and has not been optimized by incorporating the precise antioxidant nutritional requirements of pigs under different breeding scenarios, stress conditions and physiological stages. In addition, key industrial application issues, such as the toxicity threshold of excessive VA supplementation, the secondary oxidative stress damage caused by long-term low VA intake, the cost-benefit characteristics of different VA preparations, and the optimal compatibility ratio between VA and other active substances, have not been clearly clarified. As a result, existing research findings cannot provide sufficient support for the practical application of precision antioxidant nutrition regulation technology in large-scale pig farms.

Against the backdrop of the existing research gaps and the high-quality development requirements of the modern swine industry, future research on the antioxidant regulatory mechanism of VA can be carried out from the following four core directions: First, standardized animal experimental models should be constructed, with unified definitions of core experimental parameters such as breed, age, basal diet composition and stress conditions, so as to systematically verify the regulatory effect of VA on controversial antioxidant endpoints and establish quantitative regulatory criteria for different physiological stages. Second, by means of integrated multi-omics strategies including transcriptomics, proteomics and metabolomics, the core signaling pathways, target genes and molecular regulatory networks of VA regulating redox homeostasis in pigs can be deeply explored, so as to clarify the tissue-specific regulatory characteristics of VA and the cross-regulation mechanism among oxidation, immunity and intestinal barrier function. Third, a three-dimensional precise regulation model integrating VA dosage, preparation type and administration timing should be constructed for different breeding scenarios (conventional breeding, heat stress breeding, high-density breeding), so as to establish industrially applicable antioxidant nutrition standards. Fourth, efficient compound preparations composed of VA and natural antioxidant extracts, probiotics and trace elements should be developed, and low-cost and residue-free nutritional intervention technologies for relieving pig stress should be formed, so as to provide support for the sustainable healthy development of the swine industry.

## 6. Conclusions

VA regulates porcine redox homeostasis through multiple coordinated mechanisms, including direct free radical scavenging, transcriptional regulation of antioxidant-related genes, blocking the vicious cycle between inflammation and oxidative stress, and maintaining the structural stability of biological membranes. Beyond its antioxidant function, the biological effects of VA supplementation in pigs are regulated by prominent stress-dependent characteristics, dose-response patterns, feed formulation properties and nutrient interactions. VA exerts stage-specific physiological effects in weaned piglets, growing-finishing pigs, breeding sows and breeding boars when provided at physiological nutritional doses. In weaned piglets, adequate VA supplementation can alleviate oxidative damage induced by weaning stress, maintain the structural integrity of the intestinal barrier and reduce the risk of diarrhea, but excessive VA intake will induce lipotoxicity and aggravate oxidative injury. For growing-finishing pigs, in addition to activating the muscle antioxidant defense system and increasing GSH concentration, dietary VA can also regulate lipid peroxidation and meat color stability of pork after slaughter; however, supra-nutritional VA may compete with vitamin E for tissue deposition and impair the storage quality of pork. For breeding sows, most existing reproductive-related studies focus on intramuscular injection of β-carotene or VA, while relevant data obtained from practical dietary supplementation is still insufficient. Maternal VA supply may regulate the transport of retinol via placenta and colostrum-milk, and further affect the antioxidant status and early development of offspring, but consistent dose-response evidence is still lacking. Current research on the antioxidant function of VA in breeding boars is still in the exploratory stage.

The in vivo efficacy of VA is further modulated by complex crosstalk among different nutrients. When VA is combined with vitamin E, vitamin C and selenium, synergistic antioxidant effects can be observed; in contrast, excessive intake of dietary vitamin D, copper or zinc will inhibit intestinal absorption of VA, reduce its bioavailability, and thereby weaken its physiological benefits. Although relevant experimental evidence has been accumulated gradually, many ambiguities regarding the underlying mechanism remain unresolved: most of the current transcriptional evidence for RAR-mediated activation of antioxidant genes comes from in vitro cell experiments, and direct in vivo molecular verification in pigs remains insufficient. In addition, the context-dependent conflicting crosstalk between RAR and Nrf2 also requires further in-depth investigation.

From the perspective of actual swine production, the existing feeding standards for VA are primarily formulated based on growth performance indicators, rather than antioxidant-specific requirements under different stress conditions. This status quo forms a key constraint for the implementation of precision antioxidant nutrition regulation in commercial pig farms. Future research should prioritize the following directions: constructing standardized stress animal models, clarifying the tissue-specific functions of VA receptor subtypes, elucidating the dose-dependent effects of graded dietary VA intake on maternal-offspring retinol transfer, and optimizing appropriate dosage forms and compatible nutrient combinations adapted to actual breeding scenarios. The application of multi-omics technical approaches will contribute to addressing the currently unresolved mechanistic questions and provide theoretical support for developing targeted nutritional strategies to improve stress resistance, production performance, pork quality and reproductive efficiency in pig herds.

## Data Availability

No new data were created or analysed in this study. Data sharing is not applicable to this article.
